# Two new species of the stonefly genus *Amphinemura* (Insecta, Plecoptera, Nemouridae) from China

**DOI:** 10.3897/zookeys.404.7067

**Published:** 2014-04-22

**Authors:** Xiao-Yu Ji, Yu-Zhou Du, Zhi-Jie Wang

**Affiliations:** 1 School of Horticulture and Plant Protection & Institute of Applied Entomology,; 2 Yangzhou University, Yangzhou, 225009, China; 3 Suqian College, Suqian, Jiangsu, 223800, China

**Keywords:** *Amphinemura*, Nemouridae, Plecoptera, new species, China

## Abstract

Two new species of the genus *Amphinemura* Ris from China are described and illustrated, i.e. *A. annulata* Du & Ji, **sp. n.** from Zhejiang, Shanxi, Shaanxi and Guizhou Province, and *A. lingulata* Du & Wang, **sp. n.** from Shaanxi and Sichuan Province. *A. annulata* is similar to *A. tricintusidens* Wang & Zhu in having an apical cavity of the epiproct, but the epiproct ventral sclerite and the median paraproct lobe of the two species are different. *A. lingulata* is related to *A. didyma* Zhu & Yang in having the similar epiproct, but they differ mostly in paraproct median and outer lobes.

## Introduction

The subfamily Amphinemurinae includes seven genera, i.e. *Amphinemura* Ris, *Indonemoura* Baumann, *Mesonemoura* Baumann, *Protonemura* Kempny, *Sphaeronemoura* Shimizu & Sivec, *Malenka* Ricker and *Tominemoura* Sivec & Stark, and the first five genera were found in China. The genus *Amphinemura* is the largest genus of Amphinemurinae with more than 170 species from the Oriental and Holarctic Regions (Baumann 1975; DeWalt et al. 2013). The *Amphinemura* in China is represented by at least 70 species (Du et al. 2007; Du and Wang 2007; Li et al. 2005; Li and Yang 2005, 2006, 2007, 2008a, b, c, d, e, 2011, 2013; Wang et al. 2006; Wang et al. 2007; Wu 1926, 1935, 1938, 1949, 1962, 1973; Yang et al. 2005; Yang et al. 2005 and Zhu and Yang 2002, 2003). Herein we describe two new Chinese species of *Amphinemura* based on male specimens.

## Materials and methods

All type specimens are preserved in 75% or 99% ethanol and are deposited at the School of Horticulture and Plant Protection & Institute of Applied Entomology, Yangzhou University, China. Specimens were examined and illustrated using a Leica stereomicroscope-MZAPO. Abdomens were cut from the bodies, then treated in 5% NaOH, slowly heated to 40–50 °C for 1–3 minutes, and then the specimens were cleared rinsing in clean water. The morphological terminology follows that of Baumann (1975).

## Results

### Amphinemura annulata

Du & Ji sp. n.

http://zoobank.org/F99B5974-7245-4B96-A61C-147FA0A25B4E

http://species-id.net/wiki/Amphinemura_annulata

Figs 1–6

#### Material examined.

Holotype ♂ from China, Zhejiang Province, Mt. Tianmu, 300m, 18-20 Mar. 2006, leg. Wang Zhi-Jie, Sun Yun. Paratypes 30♂♂, the same details as holotype; 1♂, Shanxi Province, Lishan Natural Reserve, 1222m, 24 Aug. 2012, leg. Shi Fu-Ming; 1♂, Shaanxi Province, Houzhenzi, Upriver of Hougou, 26 May 1995, leg. Du Yu-Zhou; 1♂, Shaanxi Province, Foping County, East River, 1240m, 25 Sept. 1996, leg. Xing Lian-Xi; 1♂, Guizhou Province, Maolan City, Dongkuang, 22 Oct. 1996, leg. Li Zi-Zhong.

#### Adult habits.

Head and antennae brown, pronotum light brown, subquadrate, angles bluntly rounded, anterior margin wider than posterior margin, with lightly rugosities. Legs brown. Wings hyaline, light brown, veins brown.

#### Male.

**Figures 1–6.**
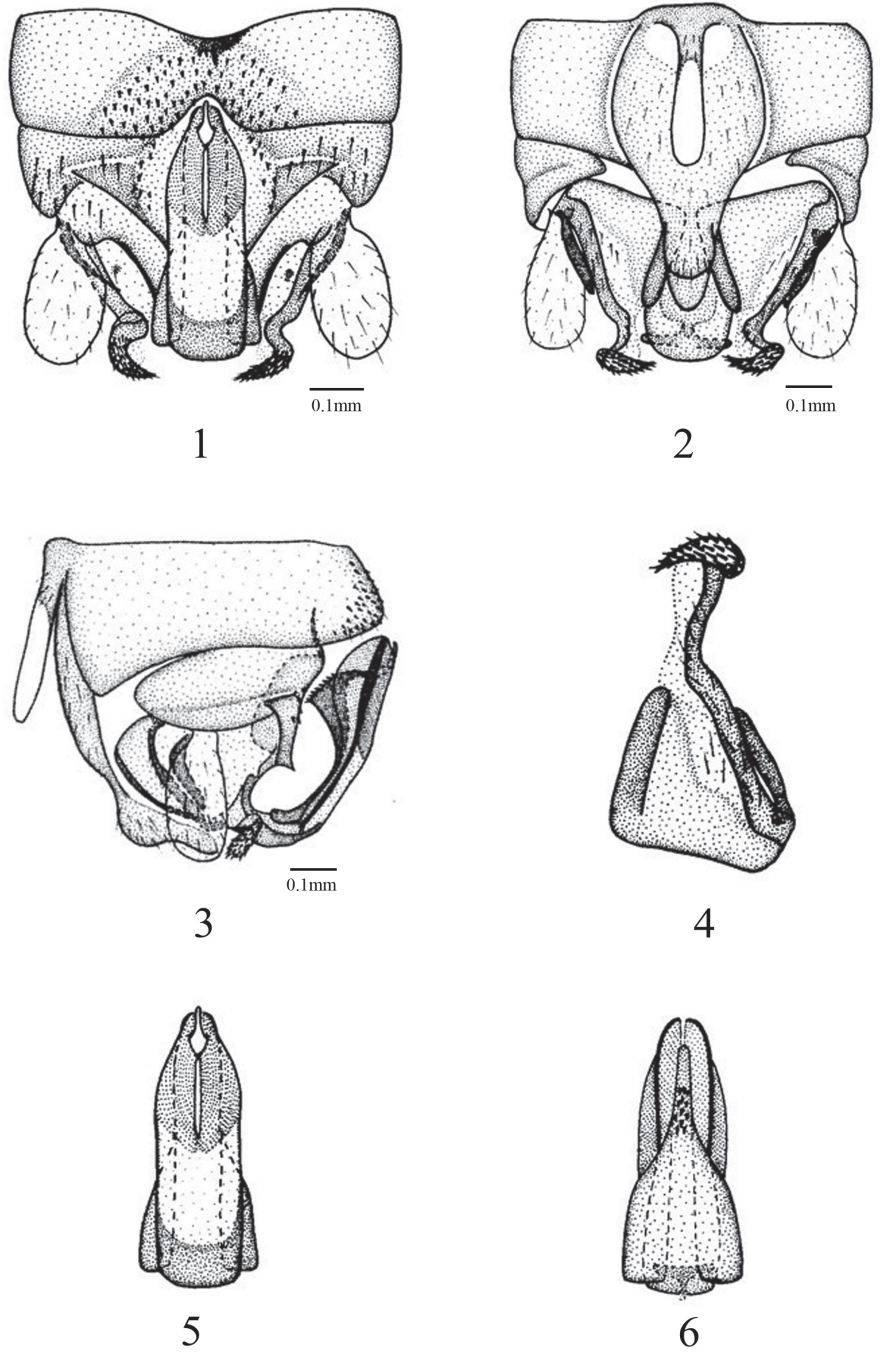
*Amphinemura annulata* male structures. **1** terminalia, dorsal aspect **2** terminalia, ventral aspect **3** terminalia, lateral aspect **4** paraproct (left) **5** epiproct, dorsal aspect **6** epiproct, ventral aspect.

Forewing length 7.7–8.2 mm, hind wing length 6.6–6.8 mm. Tergum 9 weakly sclerotized, with a small mid-posterior indention, and bearing a large bundle of tiny spines medially. Tergum 10 weakly sclerotized, with a rounded concavity below epiproct, bearing several spines on lateral margin, and a small triangular projections extruding from the base of the concavity, which is more distinct in lateral view (Figs 1 and 3). Hypoproct narrow basally, extending at midpoint and tapering with a blunt rounded tip, bulging before apex, which is more distinct in lateral view; vesicle slender, four times longer than wide (Figs 2 and 3). Paraproct divided into 3 lobes; inner lobe weakly sclerotized, thin and long, about half length of median lobe; median lobe broad basally, mostly membranous, with a long sclerotized band in its outer margin, apically curved inwards and forming an annular projection, bearing many tiny dense spines on the projection; outer lobe sclerotized, short, equal length to inner lobe (Fig. 4). Epiproct elongated, dorsal sclerite sclerotized basally, apical portion weakly sclerotized, taking up half of the dorsal sclerite, forming a small cavity at tip (Fig. 5); in lateral view, slender, well sclerotized pair of large lobes extending to ventral surface from midpoint (Fig. 3); ventral sclerite forming a triangular keel, which is more distinct in lateral view, bearing several black spines ventrally, tip of the ventral sclerite rising from the tip cavity of dorsal sclerite (Fig. 6).

#### Female.

Unknown.

#### Etymology.

The Latin “*annulata*” refers to median lobe of paraproct forming an annular projection pointing inwards.

#### Diagnosis.

This new species is similar to *Amphinemura tricintusidens* Wang & Zhu, 2007 (in Wang et al. 2007). Both species having the dorsal sclerite of the epiproct with an apical cavity, but the new species can be separated from the latter by the form of the ventral sclerite of the epiproct and the median lobe of the paraproct. In *Amphinemura annulata* sp. n., the ventral sclerite forming a triangular keel, bearing several black spines ventrally, whereas in *Amphinemura tricintusidens*, the ventral sclerite of the epiproct forms two heavily sclerotized sclerites which fused at the apex, each bearing two rows of denticles on the surface of the sclerotized sclerites. The median paraproct lobe of *Amphinemura annulata* sp. n. is mostly membranous, its outer margin sclerotized to form a long sclerotized band, and forming an annular projection pointing inwards bearing many tiny dense spines on the projection, In *Amphinemura tricintusidens*, the median lobe is sclerotized, tapering medially, with a darkly sclerotized rounded tip at the apex, and 3 or 4 dentations surrounding one side of the tip.

### Amphinemura lingulata

Du & Wang sp. n.

http://zoobank.org/DC92AC76-9A64-482E-887A-4C3D2F594D46

http://species-id.net/wiki/Amphinemura_lingulata

Figs 7–12

#### Material examined.

Holotype ♂ China, Shaanxi Province, Houzhenzi, Upriver of Hougou, 26 May 1995, leg. Du Yu-Zhou. Paratypes 6♂♂, Sichuan Province, Laohegou Natural Reserve, 1700m, 25 May 2012, leg. Ji Xiao-Yu, Tang Xiao-Tian; 2♂♂, Shaanxi Province, Qinling Mountain Range, Railway Station of Qinling, 15 May 1995, leg. Wang Min.

#### Adult habitus.

Head brown, antennae light brown, pronotum brown, with light rugosities. Legs brown. Wings subhyaline, light brown, veins brown.

#### Male.

**Figures 7–12.**
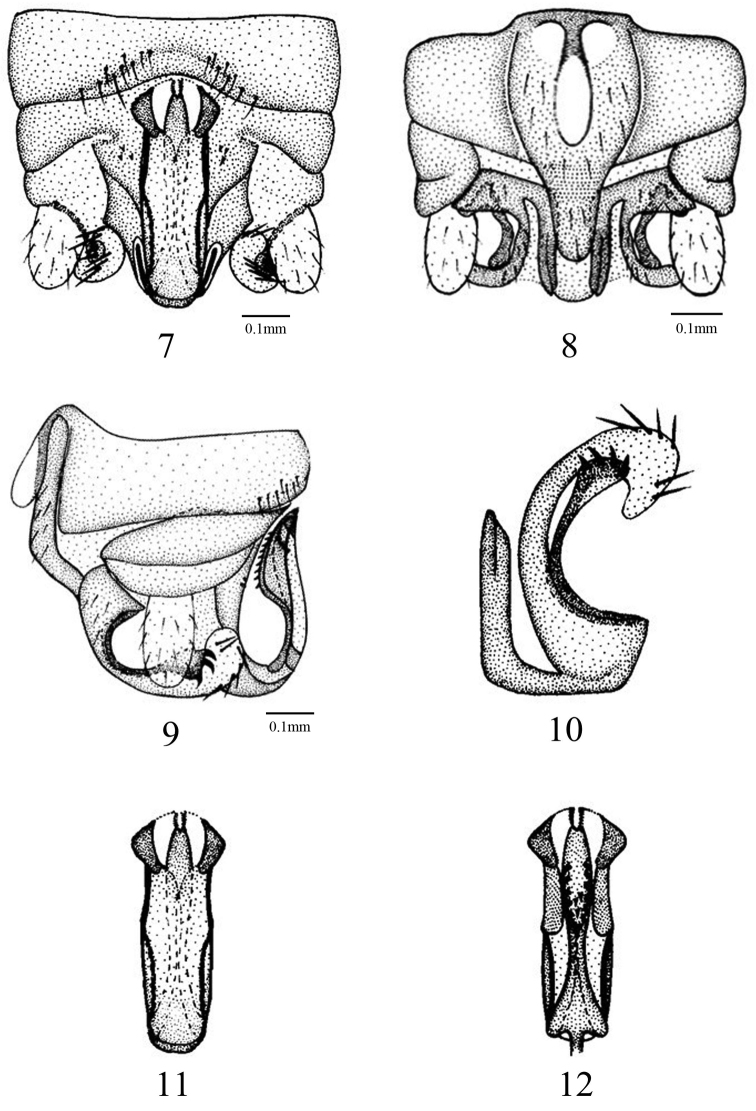
*Amphinemura lingulata* male structures. **7** terminalia, dorsal aspect **8** terminalia, ventral aspect **9** terminalia, lateral aspect **10** paraproct (left) **11** epiproct, dorsal aspect **12** epiproct, ventral aspect.

Forewing length 6.5–6.8 mm, hind wing 5.4–5.8 mm. Tergum 9 weakly sclerotized, bearing a row of long hairs at distal margin. Tergum 10 weakly sclerotized, with a large flat area below the epiproct, with few spines each side of the epiproct (Fig. 7). Hypoproct broad basally and tapering toward blunt rounded tip, vesicle slightly constricted basally, three times longer than wide (Fig. 8). Paraproct divided into 3 lobes; inner lobe weakly sclerotized, slender, with a short darkly sclerotized line medially; median lobe weakly sclerotized basally, bearing several long strong spines on the large membranous and strongly curved tip; outer lobe darkly sclerotized, slender, with 4 or 5 strong spines at the triangular tip (Fig. 10). Epiproct slender in the dorsal aspect; dorsal sclerite side mostly membranous, with a pair of sclerotized small triangular tongue-shaped projections encasing each side of the bifurcated tip (Fig. 11); lateral arms slender, darkly sclerotized, a pair of sclerotized lateral bands convergent apically (Fig. 9); ventral sclerite forming a median keel-shaped ridge, bearing many black spines ventrally (Fig. 12).

#### Female.

Unknown.

#### Etymology.

The Latin “*lingulata*” refers to the pair of small triangular tongue-shaped projections encasing each side of the tip of epiproct.

#### Diagnosis.

This new species is related to *Amphinemura didyma* Zhu & Yang (2002) in having the similar median and outer paraproct lobe. However, their epiprocts are markedly different. *Amphinemura lingulata* sp. n. is also similar to *Amphinemura zhoui* Li & Yang (2008b), *Amphinemura helanshana* Li, Murányi & Yang (2013) and *Amphinemura tibetensis* Zhu & Yang (2003) in the epiproct with a pair of sclerotized small projections encasing each side of the tip, but their paraproct lobes are different obviously.

## Concluding remarks

Shimizu (1996), in a PhD study on East Asian Nemouridae, suggested seven species-groups for East Asian species of *Amphinemura*, i.e. the *flavicollis* group, the*clavigera* group, the *spinigera* group, the *sagittata* group, the *flavostigma* group, the *pentagona* group and the*megaloba* group, and arranged some Chinese species in these species-groups. However, only the *flavostigma* group and the *megaloba* group were subsequently published (Shimizu 1998a, b). A revision of the genus *Amphinemura* will be needed considering the high number of species included.

So far 74 species of *Amphinemura*, including the two new species documented in this paper, were recorded in China. It can be expected that more *Amphinemura* species will be found in the future because China is across the Palaearctic and Oriental Region and features not yet faunistically explored areas with suitable resources for stoneflies. Additionally, studies focused on the biogeography, phylogeny and evolution are also needed to further understand the species diversity of *Amphinemura*.

## Supplementary Material

XML Treatment for Amphinemura annulata

XML Treatment for Amphinemura lingulata
